# MINNESOTA’S ASSISTED LIVING LICENSE: ENGAGEMENT OF SMALL ASSISTED LIVING PROVIDERS

**DOI:** 10.1093/geroni/igae098.0503

**Published:** 2024-12-31

**Authors:** Rajean Moone, Tetyana Shippee, Megan Hakanson

**Affiliations:** University of Minnesota, St. Paul, Minnesota, United States; University of Minnesota School Of Public Health, Minneapolis, Minnesota, United States; University of Minnesota, Minneapolis, Minnesota, United States

## Abstract

When Minnesota passed State Statute 144G in 2019 it fundamentally changed the licensure of assisted living communities in the state. This new regulatory framework was developed by a group of stakeholders representing consumer advocates, provider advocates, and state government. However, assisted living providers who served culturally specific persons in small communities were largely absent from the development of this new license. As a result, several unintended consequences and challenges have emerged in implementation in these settings. We interviewed a sample of 14 licensed assisted living directors operating small, culturally specific communities to understand their experiences with the regulatory framework. After a qualitative analysis four main themes emerged: (1) lack of participation in the initial license development, (2) challenges to implementation of license requirements, (3) inconsistency in and preparation for survey inspections, and (4) inadequate reimbursement and funding. To address these concerns, several recommendations were identified which ranged from ensuring all stakeholders are engaged in policy development to developing a new rate setting methodology for Medicaid waivers. Although these recommendations are specific to Minnesota, they do have implications for other states and agencies that are developing similar regulations.

